# Development of a Humanized Antibody Targeting Extracellular HSP90α to Suppress Endothelial-Mesenchymal Transition-Enhanced Tumor Growth of Pancreatic Adenocarcinoma Cells

**DOI:** 10.3390/cells13131146

**Published:** 2024-07-04

**Authors:** Chi-Shuan Fan, Hui-Chen Hung, Chia-Chi Chen, Li-Li Chen, Yi-Yu Ke, Teng-Kuang Yeh, Chin-Ting Huang, Teng-Yuan Chang, Kuei-Jung Yen, Chung-Hsing Chen, Kee Voon Chua, John Tsu-An Hsu, Tze-Sing Huang

**Affiliations:** 1 National Institute of Cancer Research, National Health Research Institutes, Miaoli 35053, Taiwan; change0935693367@gmail.com (C.-S.F.); poseking2430@nhri.org.tw (C.-C.C.); lilichen@nhri.org.tw (L.-L.C.); chchen@nhri.edu.tw (C.-H.C.); prisckv9047@gmail.com (K.V.C.); 2 Institute of Biotechnology and Pharmaceutical Research, National Health Research Institutes, Miaoli 35053, Taiwan; huichen@nhri.edu.tw (H.-C.H.); yiyuke@dcb.org.tw (Y.-Y.K.); tkyeh@nhri.edu.tw (T.-K.Y.); cthuang@nhri.edu.tw (C.-T.H.); tychang@nhri.edu.tw (T.-Y.C.); a-guei@nhri.edu.tw (K.-J.Y.); tsuanhsu@nhri.edu.tw (J.T.-A.H.); 3 Anbogen Therapeutics, Taipei 11571, Taiwan; 4 Department of Biochemistry, School of Medicine, Kaohsiung Medical University, Kaohsiung 80708, Taiwan; 5 Doctoral Program in Tissue Engineering and Regenerative Medicine, Biotechnology Center, National Chung Hsing University, Taichung 40227, Taiwan

**Keywords:** extracellular HSP90α, CD91, endothelial-mesenchymal transition, M2 macrophage, tumor immunity

## Abstract

Extracellular HSP90α (eHSP90α) is a promoter of tumor development and malignant progression. Patients with malignancies, including pancreatic ductal adenocarcinoma (PDAC), have generally shown 5~10-fold increases in serum/plasma eHSP90α levels. In this study, we developed a humanized antibody HH01 to target eHSP90α and evaluated its anticancer efficacy. HH01, with novel complementarity-determining regions, exhibits high binding affinity toward HSP90α. It recognizes HSP90α epitope sites _235_AEEKEDKEEE_244_ and _251_ESEDKPEIED_260_, with critical amino acid residues E237, E239, D240, K241, E253, and K255. HH01 effectively suppressed eHSP90α-induced invasive and spheroid-forming activities of colorectal cancer and PDAC cell lines by blocking eHSP90α’s ligation with the cell-surface receptor CD91. In mouse models, HH01 potently inhibited the tumor growth of PDAC cell grafts/xenografts promoted by endothelial-mesenchymal transition-derived cancer-associated fibroblasts while also reducing serum eHSP90α levels, reflecting its anticancer efficacy. HH01 also modulated tumor immunity by reducing M2 macrophages and reinvigorating immune T-cells. Additionally, HH01 showed low aggregation propensity, high water solubility, and a half-life time of >18 days in mouse blood. It was not cytotoxic to retinal pigmented epithelial cells and showed no obvious toxicity in mouse organs. Our data suggest that targeting eHSP90α with HH01 antibody can be a promising novel strategy for PDAC therapy.

## 1. Introduction

Heat Shock Protein 90α (HSP90α) has been considered to be a cancer therapeutic target for more than 20 years due to its prevalent overexpression in human cancer cells and its function as a chaperone that aids in the folding, maturation, and trafficking of several tumor-promoting proteins such as HIF-1α, mutated p53, STAT-3, CDK4, Akt, Bcr-Abl, and ErbB2/Neu [1,2,3]. Thus far, at least 18 small-molecule inhibitors have been evaluated in clinical trials, but none of them has received approval from the U.S. Food and Drug Administration for formal clinical use, mainly due to their toxic adverse effects [3,4]. Actually, HSP90 is abundantly expressed, constituting 2~3% of total cellular proteins in normal cells. In cancer cells, its expression increases by only 2~3-fold compared to normal cells [5]. This minor druggable distinction renders the uses of HSP90 inhibitors easy to reach toxic dosages in normal hepatocytes, erythrocytes, and retinal pigmented epithelial cells [2,3,4]. However, HSP90α can be secreted by tumor cells, especially in unfavorable microenvironments [6,7]. The secreted extracellular HSP90α (eHSP90α) can function as an inducer of tumor cell epithelial-mesenchymal transition (EMT), migration, invasion, and metastasis through binding to the cell-surface receptor CD91 [7,8,9]. Clinically, the plasma/serum levels of eHSP90α have been detected to increase by 5~10-fold in several malignancies, including non-small cell lung cancer, colorectal cancer (CRC), and pancreatic ductal adenocarcinoma (PDAC) [6,7,10,11], implying that eHSP90α can be a better candidate as a cancer therapeutic target.

Elevated eHSP90α levels have also been detected in serum specimens from pancreatitis patients and LSL-K-Ras^G12D^/Pdx1-Cre transgenic mice (KC mice) at the pre-PDAC stage, suggesting an early involvement of eHSP90α in PDAC formation [10]. The study on early-age KC mice has revealed that eHSP90α can be produced by pancreas-infiltrating myeloid-derived macrophages and stimulated pancreatic ductal epithelial cells to promote PDAC development [10]. Accumulating evidence has shown that HSP90α can also be secreted by the cells undergoing mesenchymal transition. In PDAC, 30~40% of cancer-associated fibroblasts (CAFs) can be derived from the endothelial-mesenchymal transition (EndoMT) of endothelial cells [12]. The EndoMT-derived CAFs are believed to play a tumor-promoting role because the inclusion of EndoMT-derived cells (i.e., α-SMA-expressing CD31^+^ cells) in PDAC cell grafts significantly promotes tumorigenesis as well as the recruitment of myeloid-derived macrophages [13,14]. These EndoMT cells produce eHSP90α rather than transforming growth factor (TGF)-β, interleukin (IL)-4, and IL-13 to induce macrophage M2-polarization [13]. The secreted eHSP90α facilitates the interaction of its cell-surface receptors CD91 and TLR4 on macrophages, inducing tumor-promoting M2-polarization through the MyD88-IRAK complex-associated IKKα/β−NF-κB/IRF3 and JAK2/TYK2−STAT-3 signaling pathways [15]. Moreover, eHSP90α can elicit a feedforward loop of eHSP90α production from macrophages, PDAC cells, and others, building an immunosuppressive and proangiogenic eHSP90α-rich microenvironment to enhance tumor growth and malignancy [13,14,15]. In addition, EndoMT cells can be prominently found near macrophages in the tumor specimens of CRC patients [16]. EndoMT cells can secrete HSP90α to foster CRC cell stemness and tumorigenicity [16]. Altogether, eHSP90α plays critical roles in both tumor development and malignant progression, positioning it as an important therapeutic target.

To evaluate the anti-PDAC efficacy of targeting eHSP90α, we previously used a cell-impermeable HSP90α inhibitor, DMAG-N-oxide, to treat the mice bearing PDAC cell grafts [10]. It is a water-soluble compound readily excreted out of mouse bodies. Its frequent administration indeed exhibited some inhibitory effects on the tumorigenicity and metastasis of PDAC cell grafts but simultaneously led to mouse splenic enlargement [10]. Encouragingly, nontoxic results have been obtained from our other studies. An anti-HSP90α mouse monoclonal antibody exhibited a suppressive efficacy against the EndoMT-promoted and M2-macrophage-involved PDAC tumor growth [13]. Octyl gallate (OG), a common antioxidant and preservative used safely in food additives and cosmetics, blocked the eHSP90α–TLR4 ligation on macrophages and prevented macrophage M2-polarization and more HSP90α expression induced by EndoMT-derived CAFs and secreted eHSP90α [14]. Therefore, our current study develops a humanized anti-HSP90α antibody HH01 for eHSP90α targeting. HH01 contains novel amino acid (a.a.) sequences in the complementarity-determining regions (CDRs), which exhibit high binding affinity towards eHSP90α. It is quite water-soluble but exhibits a half-life time (T_1/2_) of >18 days in the bloodstream of experimental mice, with no cytotoxic effect on retinal pigmented epithelial cells and no induction of splenic enlargement in mice. HH01 effectively blocks the binding of eHSP90α to cell-surface CD91 and suppresses invasive and spheroid-forming activities of PDAC and CRC cell lines. Evaluation of in vivo anticancer efficacy reveals that HH01 treatment can abrogate the tumor growth promoted by EndoMT-derived CAFs in subcutaneously inoculated PDAC cell grafts. Mouse serum eHSP90α levels are also suppressed as well. Moreover, HH01 treatment effectively restores immune T-cells in correlation with the reduction of M2 macrophages. These data suggest that HH01 holds promise as a valuable agent for PDAC therapy.

## 2. Materials and Methods

### 2.1. Cell Cultures

All cell cultures were conducted in a 37 °C and 5% CO_2_ humidified incubator. Mouse PDAC cell line Panc 02, human PDAC cell line BxPC-3, and human CRC cell line SW620 were cultured in RPMI-1640 medium supplemented with 10% of fetal bovine serum (FBS) and a mix of 100 units/mL penicillin, 100 μg/mL streptomycin, and 2 mM glutamine (1 × PSG). Human PDAC cell line PANC-1 cells were cultivated in Dulbecco’s Modified Eagle’s Medium (DMEM) with 10% of FBS and 1 × PSG. Under the same conditions, human CRC cell line LoVo was incubated with 20% FBS and 1 × PSG-containing Ham’s F-12 medium, and human retinal pigmented epithelial cell line ARPE-19 (ATCC CRL-2302^TM^; American Type Culture Collection, Manassas, VA, USA) was grown in ATCC-formulated DMEM:F12 medium (cat. #30-2006, ATCC) supplemented with 10% FBS and 1 × PSG. Human umbilical vein endothelial cells (HUVECs) were isolated and cultivated in M199 medium containing 20% FBS, 100 units/mL penicillin, 100 μg/mL streptomycin, and 30 μg/mL endothelial cell growth supplement (EMD Millipore, Billerica, MA, USA) [17]. For EndoMT induction, HUVECs were pre-incubated for 16 h with 2% FBS-containing M199 medium, followed by the addition of 0.3 μg/mL osteopontin (OPN) and further incubation for 24 h. Mouse endothelial cell line 3B-11 (ATCC CRL-2160^TM^) was grown in RPMI-1640 medium supplemented with 10% FBS and 1 × PSG. For EndoMT induction, 3B-11 cells were treated similarly to HUVECs except that 1% FBS-containing RPMI-1640 medium was used.

### 2.2. Mouse Models

Mouse experiments were performed with the approval of the Institutional Animal Care and Use Committee of National Health Research Institutes (NHRI-IACUC-106031-A, 109022-M2-S02, and 109196-A). To establish an EndoMT-involved tumor transplant model, 12-week-old C57BL/6 mice were subcutaneously inoculated with a mix of Panc 02 cells and EndoMT-derived CAFs (i.e., OPN-induced α-SMA-expressing CD31^+^ cells) [13]. Details of mouse treatments and schedules are provided in the figures and the related text. The sizes of developing tumors were measured superficially with a Vernier caliper, and tumor volumes were calculated with the formula 1/2 × length × width^2^. Real tumors were weighed upon mice sacrifice. To establish an EndoMT-involved tumor transplant model in humanized mice, NOD-SCID IL2R^null^ (ASID) mice transplanted with human hematopoietic stem cells (hHSC) were subcutaneously inoculated with a mix of PANC-1 cells and EndoMT-derived CAFs (i.e., OPN-treated HUVECs). The hHSC-transplanted ASID mice were obtained from the National Laboratory Animal Center (Taipei, Taiwan), and all had human CD45^+^ cell levels exceeding 38% of total lymphocytes. Subsequent mouse treatments were performed as indicated in the figure legends and the corresponding text.

### 2.3. Western Blot Analysis

The general procedure for Western blot analysis was employed to preliminarily screen the hybridomas. Mouse hybridomas were generated from mice immunized with recombinant 732-a.a. full-length human HSP90α (rHSP90α; PeproTech Co., Cranbury, NJ, USA) according to the conventional protocol [18]. The culture media of hybridomas were collected and reacted with the membrane strips containing HSP90α and molecular weight marker proteins blotted from SDS-polyacrylamide gels after electrophoreses. After washing thrice with PBS plus 0.05% Tween-20 (PBST), the membrane strips were incubated for 1 h with horseradish peroxidase-conjugated secondary antibody. Following three washes with PBST, immunoreactive protein bands were detected by enhanced chemiluminescence (Luminata^TM^ Crescendo Western HRP Substrate, EMD Millipore).

### 2.4. Enzyme-Linked Immunosorbent Assay (ELISA)

ELISA was employed to assay the HSP90α-binding activities of our prepared antibodies. Briefly, 96-well plates were coated with 12.5 ng/mL of rHSP90α in the capture buffer (BioLegend, San Diego, CA, USA). After incubation overnight at 4 °C, the coated plates were washed twice with PBST and then blocked with PBST plus 3% bovine serum albumin (BSA). The antibodies to be tested were applied and incubated for 2 h at room temperature. After three washes with PBST, horseradish peroxidase-conjugated secondary antibody was added and incubated at 37 °C for another 1 h. After three washes, 0.3 mg/mL of 3,3′,5,5′-tetramethylbenzidine in 0.015% H_2_O_2_ was added into each well and incubated in the dark at room temperature for 10 min. The reactions were stopped by the addition of 0.5 M H_2_SO_4_, and OD_450_ values were measured by an Infinite M200 microplate reader (TECAN, Männedorf, Switzerland). To assay the secreted HSP90α levels of mouse serum samples, the ELISA protocol was modified as described previously [7]. Additionally, the immunoglobulin (IgG) isotypes of our prepared anti-HSP90α antibodies were characterized using the Mouse Ig Isotyping ELISA Ready-Set-Go!™ Kit (eBioscience™, Thermo Fisher Scientific, Waltham, MA, USA).

### 2.5. Proximity Ligation Assay (PLA)

PANC-1 cells seeded onto glass coverslips at a density of 2 × 10^5^ cells per 22 × 22-mm coverslip was incubated 16 h with 0.5% serum-containing medium at 37 °C in a 5% CO_2_ humidified atmosphere. The cells were then treated with PBS or 15 μg/mL of rHSP90α in the absence or presence of 10 μg/mL of control IgG or the tested anti-HSP90α antibody for another 24-h incubation. The treated cells were fixed with 3% paraformaldehyde and blocked with the blocking solution provided in the Duolink in situ PLA kit (Olink Bioscience, Uppsala, Sweden). Furthermore, the cell samples were incubated overnight at 4 °C with anti-CD91 antibody (1:80, cat. #550495, BD Biosciences, San Jose, CA, USA) mixed with anti-HSP90α antibody (1:80, cat. #AHP-1339, AbD Serotec, Raleigh, NC, USA) or anti-IKKα antibody (1:80, cat. #3285, Epitomics Co., Burlingame, CA, USA). After washing thrice with Tris-buffered saline plus 0.05% Tween 20, the cell samples were incubated with PLA probes for the subsequent ligation and amplification procedure according to the manufacturer’s instructions for the Duolink in situ PLA kit. Finally, nuclei were counterstained with 4′,6′-diamidino-2-phenylindole (DAPI; Sigma-Aldrich Inc., St. Louis, MO, USA). Images were photographed and analyzed using a Leica TCS SP5 II confocal microscope and LAS AF Lite 4.0 software (Leica, Wetzlar, Germany).

### 2.6. Transwell Invasion Assay

In a 37 °C and 5% CO_2_ humidified incubator, cancer cells were subjected to serum starvation in 0.5% FBS-containing medium for 16 h and then added with PBS or 15 μg/mL of rHSP90α in the absence or presence of 10 μg/mL of control IgG or the tested anti-HSP90α antibody. After another 24 h, treated cells were harvested as aliquots (1 × 10^5^ cells per aliquot) in 0.5% FBS-containing culture medium, and then each aliquot was seeded into a top chamber of Transwell inserts pre-coated with 5-fold diluted Matrigel (BD Biosciences). Cells were allowed 16 h to invade through the Matrigel toward the bottom chambers containing culture medium plus 10% FBS. The filters of the Transwell inserts were then fixed and stained with Giemsa. Invasive cells on the lower side of filters were photographed using the Axiovert S100/AxioCam HR microscope system (Carl Zeiss, Oberkochen, Germany) and further quantified by the Image-Pro Plus 6.0 software (Media Cybernetics, Inc., Silver Spring, MD, USA).

### 2.7. Cell-Spheroid-Forming Assay (Cell Self-Renewal Activity Assay)

A thousand cancer cells were seeded onto each well of 24-well plates pre-coated with a 4-mm thick layer of 0.5% agarose. After incubation for 24 h with a serum-free medium at 37 °C in a 5% CO_2_ humidified atmosphere, cells were added with PBS or 15 μg/mL of rHSP90α in the absence or presence of 10 μg/mL of control IgG or the tested anti-HSP90α antibody. The incubation was kept for 10~14 days with supplements of some fresh serum-free medium every 3 days. Finally, the cell spheroids characterized by tight, non-adherent, and >100 μm in diameter were photographed and counted under an Olympus IX 71 inverted microscope (Center Valley, PA, USA). The spheroid formation % was calculated by the formula: 100% × number of cell spheroids/1000.

### 2.8. Cloning and Sequencing of Mouse Antibody Gene from Hybridoma Cells

Total RNA was isolated from anti-HSP90α IgG-producing mouse hybridoma cells and converted to cDNA by reverse transcriptase plus random hexamers. Mouse Ig-Primer Set (Sigma-Aldrich) was used for the PCR amplification of the variable domains of IgG heavy (H) and light (L) chains (i.e., V_H_ and V_L_ domains). The PCR products were inserted into the pJET1.2/blunt cloning vector supplied in the CloneJET PCR cloning kit (Thermo Fisher Scientific), followed by the general procedure of clone selection and DNA sequence analysis.

### 2.9. Construction and Expression of Recombinant Antibodies

The cDNA of the IgG V_H_ and V_L_ domains were synthesized by GeneDireX, Inc. (Taoyuan, Taiwan) and inserted into pFUSEss-CHIg-hG1e1 and pFUSE2ss-CLIg-hk plasmids (InvivoGen, San Diego, CA, USA), respectively, at the sites just before the regions encoding the constant domains of H and L chains (i.e., C_H_ and C_L_ domains). The V_H_ cDNA was inserted into pFUSEss-CHIg-hG1e1 plasmid at the *Eco*R I-*Nhe* I site behind the human IL-2 signal sequence to express a secreted recombinant protein containing the Fc fragment with mutations at Q250T, E356D, M358L, and L428M residues. On the other hand, the cDNA of the V_L_ domain, after digestion with *Eco*R I and *Bsi*W I, was inserted into a pFUSE2ss-CLIg-hk plasmid. The recombinant DNA sequences encoding the recombinant IgG H and L chains were further amplified using hIgHG-F/hIgHG-R and CLIg-F/CLIg-R primers and then inserted into pcDNA3.4-TOPO plasmid (Thermo Fisher Scientific), respectively. Finally, ExpiCHO cells were transfected with a mix of H and L-chain-encoding plasmids at a 2:3 molar ratio. The media of transfected ExpiCHO cells were harvested after culture for 8–10 days. The recombinant antibody was purified by protein A columns of the Gibco^TM^ ExpiCHO^TM^ Expression System (Thermo Fisher Scientific).

### 2.10. Antibody Humanization and Optimization

The F(ab’)2 regions of mouse antibody were subjected to humanization with the aid of computational methods, and all calculations were performed by the Discovery Studio 2018 software (BIOVIA Inc., San Diego, CA, USA). The CDRs were identified by the published method [19]. The CDRs of mouse antibodies were grafted into in-house human templates for further three-dimensional (3D)-structure calculations. Computer modeling was conducted using the X-ray templates of PdbID: 4X0K and PdbID: 4Y5X [20,21]. The calculations included the aspects of aggregation, post-translational modification, protease cleavage, pharmacokinetics, and stability for the mutation suggestions to improve the recombinant antibody.

### 2.11. Cloning, Expression, and Purification of Full-Length and Truncated HSP90α

The full-length HSP90α (a.a. 1~732) and its 3 truncated fragments, including the N-terminal plus Linker domains (a.a. 1~272), N terminus through Middle domain (a.a. 1~629), and C-terminal domain (a.a. 630~732) were cloned, expressed, and purified. Briefly, each cDNA sequence with an 8 × His-tag was amplified by PCR from the human ORF Clone (NM_005348) with the primers containing *Xho* I and *Bam*H I sites. The PCR products were purified, digested with restriction enzymes, and then inserted into pET-23a plasmid (Sigma-Aldrich). The resultant recombinant plasmids were used for the transformation of *Escherichia coli* ClearColi^®^ competent cells (Lucigen Co., Middleton, WI, USA). The *E. coli* transformants were then stimulated with isopropyl-D-thiogalacto-pyranoside to express the respective recombinant constructs, which were further purified by the Ni-NTA column (Qiagen, Germantown, MD, USA) according to the manufacturer’s instructions. The purified proteins were finally dialyzed against the storage buffer consisting of 25 mM Tris-HCl (pH, 7.0), 50 mM NaCl, 0.1% Triton X-100, 50% glycerol, 1 mM EDTA, 1 mM DTT, and protein concentrations were determined by the Bradford protein assay (Bio-Rad Laboratories, Hercules, CA, USA).

### 2.12. Epitope Determination

Initially, the domain scanning strategy was used to broadly delineate the HSP90α region responsible for the binding with our prepared antibodies. The full-length HSP90α and its 3 truncated fragments were employed to coat 96-well ELISA plates for assessing the binding activities of the mouse monoclonal antibodies under test. The results suggested that the tested mouse monoclonal antibody bound to the a.a. 1~272 region of HSP90α. Therefore, in the peptide scanning strategy, a library containing 132 peptides was synthesized by Mimotopes Pty. Ltd. (Melbourne, VI, Australia), which comprised a series of 10-a.a. peptides offset by 2 a.a. to span the sequence of the a.a. 21~272 region of HSP90α. These peptides were used to coat 96-well ELISA plates to assess the binding activities of the antibodies of interest. To monitor the specificity of peptide-antibody interactions, secondary antibodies alone were also added and reacted with all peptides coated on the plates, and the OD_450_ readings were measured at the background level (ranging from 0.06 to 0.11). Based on the sequences suggested by the peptide scanning result, the alanine scanning strategy was further employed to investigate which a.a. residues were critical for the binding of the tested antibodies. The result of the peptide scanning assay suggested _235_AEEKEDKEEE_244_ and _251_ESEDKPEIED_260_ of HSP90α as the epitopes for binding with the tested antibodies, and therefore, 19 peptides were synthesized with sequential substitution of each a.a. by alanine (Genozyme Biotech Inc., Taipei, Taiwan). These peptides were subjected to ELISA as described above.

### 2.13. HSP90α-Binding Affinity Assay

To measure the HSP90α-binding characteristics of anti-HSP90α antibodies, binding kinetic assays were performed using Biacore T200 (Cytiva Co., Marlborough, MA, USA). The anti-HSP90α antibodies to be tested were applied onto Protein A Series S sensor chips as ligands to achieve approximately 3000~5000 response units. A series of concentrations of rHSP90α (1.5625~100 nM) in HBS-EP+ buffer was injected over the chip surface. The association was monitored for 60 s, and the final dissociation time was 1500 s.

### 2.14. Retinal Cell Toxicity Assay

ARPE-19 cells were seeded onto 96-well plates at a density of 4000 cells per well and then treated in triplicate with various concentrations of 17-AAG or anti-HSP90α antibody HH01 for 72 h. The 3-(4,5-dimethylthiazol-2-yl)-5-(3-carboxymethoxyphenyl)-2-(4-sulfophenyl)-2H-tetrazolium (MTS; Sigma-Aldrich) assay was employed and OD_490_ was measured to evaluate cell viability upon each treatment.

### 2.15. Pharmacokinetic Assay

The pharmacokinetic profile of anti-HSP90α antibody HH01 was studied in male Bltw:CD1(ICR) mice (BioLASCO Taiwan Co., Taipei, Taiwan). Mice (n = 5) with a body weight of 25~30 g per mouse were intravenously injected with 10 mg/kg of HH01 antibody. Food and water were available ad libitum at all times. For determination of the serum exposure levels of HH01 antibody, blood samples were collected at Day-0.04, 0.08, 0.25, 1, 2, 3, 7, 9, 14, 16, 18, 21, 23, 25, 35, 42, 49, 56, and 63 post-administration. These samples were further measured for the human IgG1 levels by an ELISA kit (Cayman Chemical Co., Ann Arbor, MI, USA) to represent the remaining HH01 levels in mouse sera.

### 2.16. Immunohistochemical Staining

Mouse tissue sections with a 4-μm thickness were deparaffinized by xylene and rehydrated in graded ethanol dilutions. Antigen retrieval was performed by heating the sections for 15 min in 10 mM of citrate buffer at pH 6.0 under high pressure. The endogenous peroxidase activity was blocked with 0.3% H_2_O_2_. Before staining, the sections were blocked for 30 min with PBS plus 3% BSA at room temperature in a humidified chamber. Subsequently, anti-F4/80 antibody (1:100, cat. #MCA497R, AbD Serotec) or anti-CD163 antibody (1:80, cat. #sc-33560, Santa Cruz Biotechnology, Dallas, TX, USA) was applied and incubated at 4 °C for overnight. Following washing, secondary antibodies were applied and incubated for 30 min at room temperature. The sections were further detected using the DAKO REAL EnVision Detection System (Produktionsvej 42, DK-2600 Glostrup, Denmark) and counterstained with hematoxylin. The stained sections were dehydrated, mounted with mounting solution, and finally observed and photographed using the Axiovert S100/AxioCam HR microscope system.

### 2.17. Immunohistofluorescent Staining

Tissue sections from mice underwent deparaffinization, rehydration, and antigen retrieval, as detailed previously. The sections were blocked for 30 min with PBS plus 5% BSA at room temperature and then incubated overnight at 4 °C with primary antibody sets. To measure effective CD4^+^ T-cell levels, the primary antibody set included an anti-CD4 antibody (1:100, cat. #GTX44531, GeneTex Taiwan, Hsinchu City, Taiwan) and antitumor necrosis factor (TNF)-α antibody (1:75, cat. #sc-52746, Santa Cruz Biotechnology). For assaying the cytotoxic CD8^+^ T-cell levels, the primary antibody set contained anti-CD8 antibody (1:100, cat. #GTX16696, GeneTex Taiwan) and anti-TNF-α antibody (1:75, cat. #sc-52746, Santa Cruz Biotechnology). After washing, the sections were treated with the respective fluorescence-labeled secondary antibodies and further incubated for 1 h at room temperature. Nuclei were stained with DAPI. Finally, the sections were observed, photographed, and analyzed using a Leica TCS SP5 II confocal microscope and LAS AF Lite 4.0 software.

## 3. Results

### 3.1. Preparation and Characterization of Mouse Anti-HSP90α Monoclonal Antibodies

**Figure 1:**
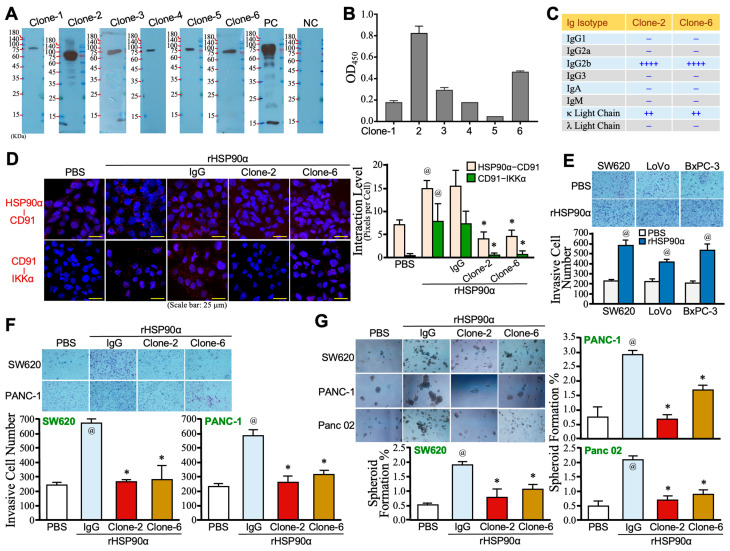
This Preparation and characterization of mouse anti-HSP90α monoclonal antibodies. (**A**) Western blotting analysis of HSP90α-binding abilities of culture supernatants from 6 hybridoma clones derived from mice immunized with human full-length rHSP90α. Control IgG was used as a negative control (NC), and a commercially available anti-HSP90α antibody (#GTX109753, GeneTex Inc., Hsinchu City, Taiwan) was used as a positive control (PC). (**B**) ELISA results showing HSP90α-binding activities in culture supernatants of the six hybridoma clones. (**C**) Subtype identification of Clone-2 and Clone-6 antibodies as IgG2b-isotype immunoglobulins containing κ light chains using the Mouse Ig Isotyping ELISA Ready-Set-Go!™ Kit (eBioscience™, Thermo Fisher Scientific). (**D**) PLA results showing physical association levels of CD91 with HSP90α and IKKα in PANC-1 cells stimulated with 15 μg/mL of rHSP90α in the absence or presence of 10 μg/mL of control IgG, Clone-2, or Clone-6 antibody. Data indicated that both Clone-2 and Clone-6 antibodies blocked ligation of eHSP90α with the cell-membrane receptor CD91 and downstream recruitment of IKKα. @ *p* < 0.05 when compared to PBS treatment. * *p* < 0.05 when compared to IgG treatment. (**E**) Transwell invasion assay showing eHSP90α-induced cancer cell invasion. SW620, LoVo, and BxPC-3 cells were serum-starved for 16 h before treatment with PBS or 15 μg/mL rHSP90α for another 24 h. Treated cells were then harvested for a 16-h Matrigel invasion assay. @ *p* < 0.05 when compared to PBS treatment. (**F**) Transwell invasion assay showing Clone-2 and Clone-6 antibodies inhibiting eHSP90α-stimulated cancer cell invasion. Serum-starved SW620 and PANC-1 cells were treated for 24 h with 15 μg/mL of rHSP90α plus 10 μg/mL of control IgG, Clone-2, or Clone-6 antibody. @ *p* < 0.05 when compared to PBS treatment. * *p* < 0.05 when compared to IgG treatment. (**G**) Cell-spheroid-forming assay showing Clone-2 and Clone-6 antibodies inhibiting eHSP90α-induced cancer cell stemness. Serum-starved SW620, PANC-1, and Panc 02 cells were treated for 24 h with 15 μg/mL of rHSP90α plus 10 μg/mL of control IgG, Clone-2, or Clone-6 antibody. Treated cells were incubated for 10~14 days for cell-spheroid formation. Cell spheroids with diameters >100 μm were counted, and spheroid formation % was calculated using the formula: 100% × (number of cell spheroids/1000). @ *p* < 0.05 when compared to PBS treatment. * *p* < 0.05 when compared to IgG treatment.

Six hybridoma clones were derived from mice immunized with human full-length rHSP90α. Culture supernatants from these clones were collected to assess their binding activities to HSP90α using standard Western blotting and ELISA protocols. All hybridoma-derived antibodies showed binding to the antigen rHSP90α (Figure 1A). The binding activities were further quantified by ELISA, and Clone-2 and Clone-6 exhibited the top two HSP90α-binding levels (Figure 1B). The antibodies from these two clones were purified and identified as IgG2b-isotype immunoglobulins containing κ light chains (Figure 1C). They efficiently blocked the binding of eHSP90α to its cell-surface receptor CD91 and the downstream association of CD91 with IKKα (Figure 1D). Given that eHSP90α promotes cancer through its stimulatory effects on cancer cell EMT, migration, invasion, and stemness acquisition, our Transwell invasion assays confirmed that rHSP90α acted as an inducer of cancer cell invasiveness (Figure 1E). This effect could be drastically antagonized by Clone-2 and Clone-6 anti-HSP90α antibodies (Figure 1F). Additionally, the rHSP90α-induced gain-of-stemness in cancer cells, as indicated by increased cell-spheroid-forming ability, could also be significantly inhibited by Clone-2 and Clone-6 antibodies (Figure 1G). Taken together, these findings suggest that anti-HSP90α monoclonal antibodies have the potential to be used as therapeutic agents against eHSP90α-promoted malignancies.

### 3.2. Humanization of Clone-2 Anti-HSP90α Antibody

**Figure 2:**
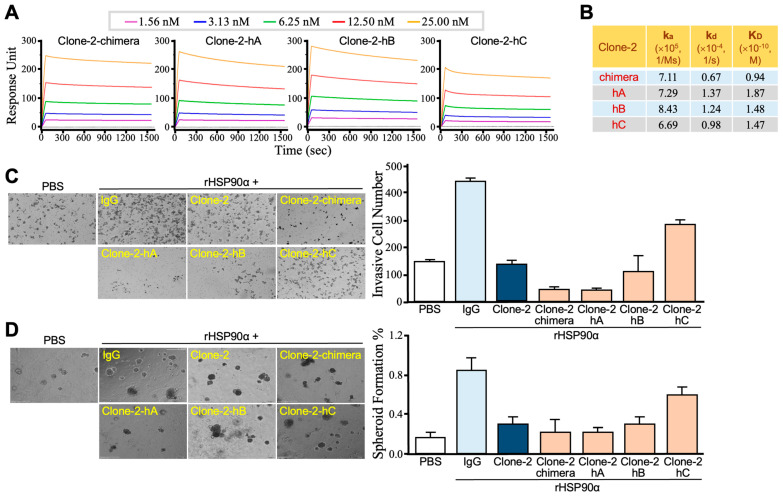
Characterization of humanized anti-HSP90α antibodies. (**A**) Binding kinetic assays showing the HSP90α-binding kinetic profiles of Clone-2-chimera, Clone-2-hA, Clone-2-hB, and Clone-2-hC anti-HSP90α antibodies. The binding sensor grams were generated using Biacore T200 and then fitted to a simple 1:1 interaction model. (**B**) The equilibrium dissociation constant (K_D_) values of Clone-2-chimera, Clone-2-hA, Clone-2-hB, and Clone-2-hC antibodies. (**C**) Transwell invasion assay showing different activities of Clone-2, Clone-2-chimera, Clone-2-hA, Clone-2-hB, and Clone-2-hC anti-HSP90α antibodies to inhibit eHSP90α-stimulated PANC-1 cell invasion. Serum-starved PANC-1 cells were treated for 24 h with 15 μg/mL of rHSP90α plus 10 μg/mL of control IgG or tested anti-HSP90α antibodies and then harvested for a 16-h Matrigel invasion assay. (**D**) Cell-spheroid-forming assay showing different activities of Clone-2, Clone-2-chimera, Clone-2-hA, Clone-2-hB, and Clone-2-hC anti-HSP90α antibodies to inhibit eHSP90α-induced PANC-1 cell stemness. Serum-starved PANC-1 cells were treated for 24 h with 15 μg/mL of rHSP90α plus 10 μg/mL of control IgG or tested anti-HSP90α antibodies and then incubated for 10~14 days for cell-spheroid formation.

Next, we cloned and analyzed the cDNA sequences of the IgG V_H_ and V_L_ domains of the Clone-2 anti-HSP90α antibody. For humanization, the V_H_ and V_L_ cDNA sequences were synthesized and inserted into plasmids at the sites just before the regions expressing the human IgG C_H_ and C_L_ domains, respectively, resulting in the expression of a recombinant IgG1 antibody called “Clone-2-chimera”. Moreover, we constructed 3 mutants of Clone-2-chimera, designated as Clone-2-hA, Clone-2-hB, and Clone-2-hC, with the goal of improving antibody properties related to aggregation, protease cleavage, post-translational modifications, pharmacokinetics, and stability, based on computer-assisted structural modeling and calculations. These engineered humanized recombinant antibodies were expressed by ExpiCHO cells and analyzed for their affinities to HSP90α after purification. Kinetics assays were performed using Biacore T200 to determine the binding characteristics of Clone-2-chimera, Clone-2-hA, Clone-2-hB, and Clone-2-hC antibodies toward rHSP90α (Figure 2A). The equilibrium dissociation constant (K_D_) values of Clone-2-chimera, Clone-2-hA, Clone-2-hB, and Clone-2-hC antibodies were further calculated as 0.94 × 10^−10^, 1.87 × 10^−10^, 1.48 × 10^−10^, and 1.47 × 10^−10^ M, respectively (Figure 2B), suggesting that the mutations in Clone-2-hA, Clone-2-hB, and Clone-2-hC antibodies did not significantly reduce the HSP90α-binding affinity of Clone-2-chimera. To preliminarily evaluate the anticancer efficacies of these humanized anti-HSP90α antibodies, we assessed their inhibitory effects on rHSP90α-induced PDAC cell invasion and spheroid formation. The Transwell invasion assay results showed that Clone-2-chimera and Clone-2-hA were more effective than Clone-2-hB, Clone-2-hC, and their parental Clone-2 in suppressing rHSP90α-induced invasiveness of human PDAC PANC-1 cells (Figure 2C). In the spheroid-forming assay, Clone-2-hA displayed the most outstanding efficacy in suppressing the rHSP90α-induced cell-spheroid formation of PANC-1 cells (Figure 2D). Consequently, Clone-2-hA was selected and named as HH01 antibody for further in vitro and in vivo assays.

### 3.3. Identification of the Epitopes of HH01 Antibody

**Figure 3:**
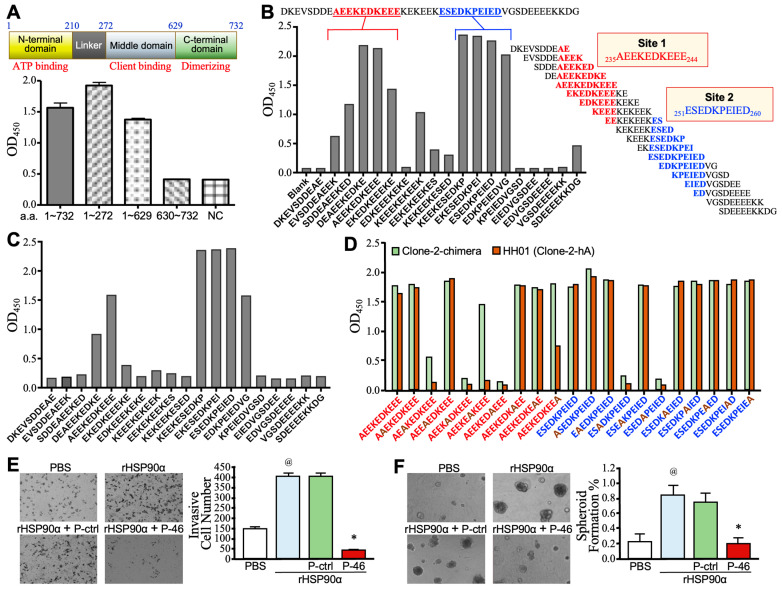
Identification of epitopes of Clone-2-chimera and HH01 anti-HSP90α antibodies. (**A**) Domain scanning assay determining which region of HSP90α bound with the Clone-2 antibody. DNA fragments of full-length HSP90α (a.a. 1~732) and its 3 truncated forms, including a.a. 1~272, a.a. 1~629, and a.a. 630~732 were cloned and expressed. The expressed proteins were purified, and their binding activities with Clone-2 antibody were assayed by ELISA. NC, buffer without sample protein as the negative control. (**B**) Peptide scanning assay investigating which sites on HSP90α bound with the Clone-2-chimera antibody. A peptide library consisting of 10-a.a. peptides with an 8-a.a. overlap between every two consecutive peptides to scan the a.a. 21~272 region of HSP90α were assayed for binding activities with Clone-2-chimera by ELISA. (**C**) Peptide scanning assay investigating which sites on HSP90α bound with HH01 antibody. The above-mentioned peptide library was assayed for binding activities with HH01 by ELISA. (**D**) Alanine scanning assay determining which a.a. residues of HSP90α were critical for binding with Clone-2-chimera and HH01 antibodies. Based on _235_AEEKEDKEEE_244_ and _251_ESEDKPEIED_260_ sites suggested by the above results, two sets of 10-a.a. peptides with a series of single a.a. residue substitutions with alanine were synthesized to assay binding activities with Clone-2-chimera and HH01 antibodies by ELISA. (**E**) Transwell invasion assay showing the activity of epitopes-containing peptide (P-46) to inhibit eHSP90α-stimulated PANC-1 cell invasion. Serum-starved PANC-1 cells were treated for 24 h with 15 μg/mL of rHSP90α along with 15 μg/mL of P-46 or control peptide (P-ctrl) and then harvested for a 16-h Matrigel invasion assay. @ *p* < 0.05 when compared to PBS treatment. * *p* < 0.05 when compared to P-ctrl treatment. (**F**) Cell-spheroid-forming assay showing the activity of P-46 to inhibit eHSP90α-induced PANC-1 cell stemness. Serum-starved PANC-1 cells were treated for 24 h with 15 μg/mL of rHSP90α along with 15 μg/mL of P-ctrl or P-46 and then incubated for 10–14 days for cell-spheroid formation. @ *p* < 0.05 when compared to PBS treatment. * *p* < 0.05 when compared to P-ctrl treatment.

HH01 can specifically bind to the human HSP90α with novel a.a. sequences in the CDRs, and thus, we aimed to identify the region(s) of HSP90α that served as the epitope(s) for HH01 recognition. In a preliminary study, we employed domain scanning to delineate the HSP90α region responsible for the binding to the Clone-2 antibody. Full-length HSP90α (a.a. 1~732) and 3 truncated fragments, including the N-terminal plus Linker domains (a.a. 1~272), N terminus through Middle domain (a.a. 1~629), and C-terminal domain (a.a. 630~732) were cloned, expressed, and purified. Subsequently, we assessed the binding activities of the Clone-2 antibody to full-length and truncated HSP90α using a conventional ELISA method. The results showed that Clone-2 antibody bound to the a.a. 1~272 region as well as the full-length protein (Figure 3A), suggesting that the binding epitope(s) of Clone-2 antibody may reside within the N-terminal plus Linker domains (a.a. 1~272) of HSP90α. Furthermore, we adopted the strategy of peptide scanning to delineate the binding epitope(s) of Clone-2-chimera and HH01 antibodies. We established a peptide library consisting of a series of 10-a.a. peptides with an 8-a.a. overlap between any two consecutive peptides to scan the sequence of the a.a. 21~272 region of HSP90α. The binding activities of Clone-2-chimera and HH01 antibodies to this series of 10-a.a. peptides were assessed by ELISA. The results revealed that the probable epitope sites of Clone-2-chimera antibody were _235_AEEKEDKEEE_244_ and _251_ESEDKPEIED_260_ of HSP90α (Figure 3B). Consistently, these two sites also served as the binding epitopes of HH01 antibody (Figure 3C). Based on the sequences of these two sites, we synthesized two series of 10-a.a. peptides with single a.a. residue substitutions with alanine. The strategy of alanine scanning was employed to investigate the a.a. residues critical for the binding of Clone-2-chimera and HH01 antibodies to the epitope sites. The results showed that the binding activities of Clone-2-chimera antibody to the epitope sites were drastically decreased when E237, E239, K241, E253, and K255 were replaced with alanine (Figure 3D). Besides, D240 was also crucial for the binding of HH01 antibody (Figure 3D). Based on the epitope mapping results mentioned above, we synthesized a peptide with a sequence of the a.a. 227~272 region of HSP90α. This peptide competitively suppressed rHSP90α-induced PANC-1 cell invasion (Figure 3E) and spheroid formation (Figure 3F), suggesting that the HSP90α epitope regions for HH01 recognition could be responsible for the binding of eHSP90α to cell-surface receptor CD91 and could be considered to be a target for developing novel anti-PDAC strategies.

### 3.4. Exhibition by HH01 of Other Characteristics Suitable for Development as a Therapeutic Agent

**Figure 4:**
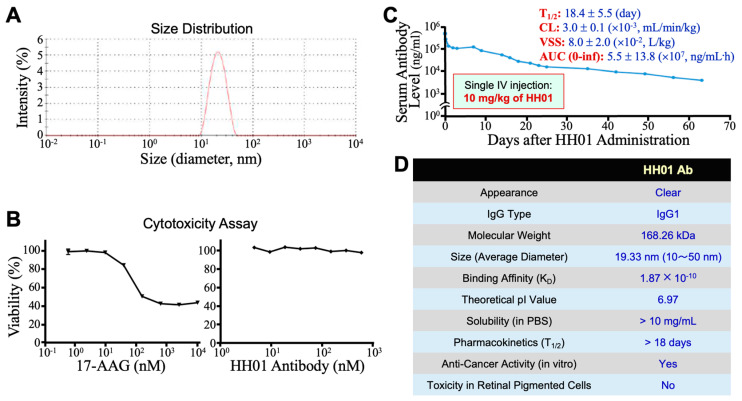
Miscellaneous characteristics of HH01 antibody. (**A**) Particle size of HH01 antibody measured using Dynamic Light Scattering. The sizes are distributed within a range of 10~50 nm, with an average diameter of 19.33 nm. (**B**) Viabilities of ARPE-19 retinal pigmented epithelial cells after 72-h treatments with different concentrations of 17-AAG and HH01 antibody, respectively. (**C**) Pharmacokinetics of HH01 antibody in the blood of male Bltw:CD1(ICR) mice (n = 5). Mice received a single intravenous (IV) injection of 10 mg/kg of HH01, and blood samples were collected at various time points post-HH01 injection to measure human IgG1 levels, representing the remaining HH01 levels in mouse sera. (**D**) Summary of some characteristics of HH01 antibody.

The solubility of HH01 in PBS was >10 mg/mL, and the Dynamic Light Scattering assay result revealed that HH01 had an average diameter of 19.33 nm within a range of 10~50 nm (Figure 4A), suggesting that HH01 did not readily form aggregates. Considering the clinical use of small-molecule HSP90 inhibitors, including geldanamycin and resorcinol derivatives, caused retinal photoreceptor cell damage when they accumulated in the retina to certain levels, we further investigated the effect of HH01 antibody on the viability of ARPE-19 retinal pigmented epithelial cells. As shown in Figure 4B, 17-AAG exhibited a cytotoxic profile to ARPE-19 cells with an IC_50_ of 64.7 nM. However, HH01 did not cause any obvious cytotoxicity in ARPE-19 cells, even at concentrations up to 594.3 nM (100 μg/mL). Additionally, the pharmacokinetics of HH01 was studied in the blood of male Bltw:CD1(ICR) mice, showing that HH01 had a T_1/2_ of 18.4 days with a clearance rate at 3 × 10^−3^ mL/min/kg and a volume in a steady state of 80 mL/kg in mouse blood (Figure 4C). Taken together, we summarized the characteristics of the HH01 antibody in Figure 4D. Besides showing anticancer activities in vitro, HH01 exhibited a long T_1/2_ and no detected cytotoxicity, strongly suggesting that HH01 is suitable for further development as a novel therapeutic agent.

### 3.5. Evaluation of HH01 Efficacy in Mouse PDAC Transplant Models

**Figure 5:**
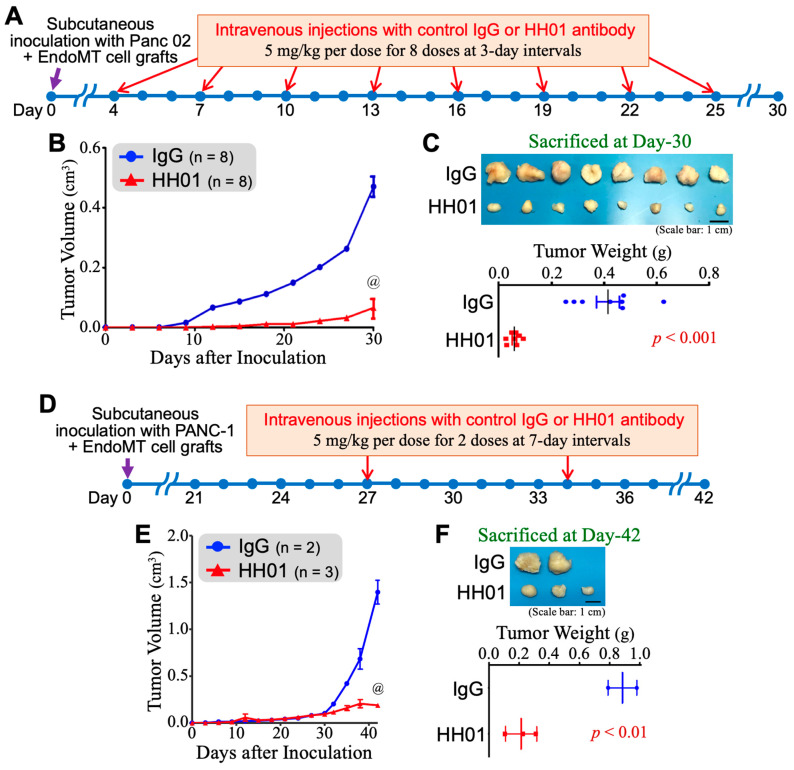
Evaluation of the anticancer efficacy of HH01 antibody in tumor transplant mouse models. (**A**) Prophylactic administration schedule of HH01 antibody to mice subcutaneously inoculated with Panc 02 plus EndoMT cell grafts. (**B**) Measurement of superficial tumor volumes of the experimental mice for plotting tumor growth curves. HH01 treatments effectively prevented tumor growth of Panc 02 plus EndoMT cell grafts. @ *p* < 0.001 when compared to IgG treatments. (**C**) Weighing of tumors surgically removed from the mice on Day 30 post-inoculation. (**D**) Therapeutic administration schedule of HH01 antibody to humanized mice subcutaneously inoculated with PANC-1 plus EndoMT cell xenografts. (**E**) Measurement of superficial tumor volumes of the humanized mice for plotting tumor growth curves. HH01 treatments effectively suppressed tumor growth of PANC-1 plus EndoMT cell xenografts. @ *p* < 0.001 when compared to the results of IgG and HH01 treatments. (**F**) Weighing of tumors surgically removed from the humanized mice on Day 42 post-inoculation.

HH01 antibody was further evaluated by in vivo anticancer assays. Given the recognized 99% identity between human and mouse HSP90α, we followed our previously reported schedule for assessing a mouse monoclonal antibody to evaluate the cancer-preventive activity of HH01 in the mouse model of EndoMT-involved PDAC. C57BL/6 mice were subcutaneously inoculated with Panc 02 cells plus EndoMT-derived α-SMA-expressing CD31^+^ cells. On post-inoculation Day 4, the mice started to receive the intravenous administration of 5 mg/kg per dose of control IgG or HH01 antibody for 8 doses at 3-day intervals (Figure 5A). As shown in Figure 5B, the tumor growth kinetics of EndoMT-involved Panc 02 cell grafts were significantly suppressed by HH01 antibody treatment. On post-inoculation Day 30, all mice were sacrificed, and their tumors were removed for weighing. As expected, HH01 treatment significantly repressed the tumor weights of Panc 02 plus EndoMT cell grafts (*p* < 0.001, Figure 5C). Furthermore, we evaluated the therapeutic efficacy of the HH01 antibody in a humanized mouse PDAC transplant model. hHSC-transplanted ASID mice were subcutaneously inoculated with a mix of human pancreatic adenocarcinoma PANC-1 cells with EndoMT-derived cells, which allowed the tumor to grow for 27 days. Because the T_1/2_ of HH01 was suggested as 18.4 days in mouse blood circulation, the mice bearing tumors of approximately 100-mm^3^ in volume started to be intravenously injected with 5 mg/kg per dose of control IgG or HH01 antibody for 2 doses at 7-day intervals (Figure 5D). Developing tumors were superficially measured every 3 days until post-inoculation Day-42 and the tumor growth curves were plotted as shown in Figure 5E. Compared with the mice treated with IgG, the mice treated with HH01 experienced significant repression of tumor growth. All mice were sacrificed on Day 42, and the tumors from the mice treated with HH01 were significantly suppressed (Figure 5F).

### 3.6. Immune Reinvigoration of Tumor Microenvironment by HH01 Treatment

**Figure 6:**
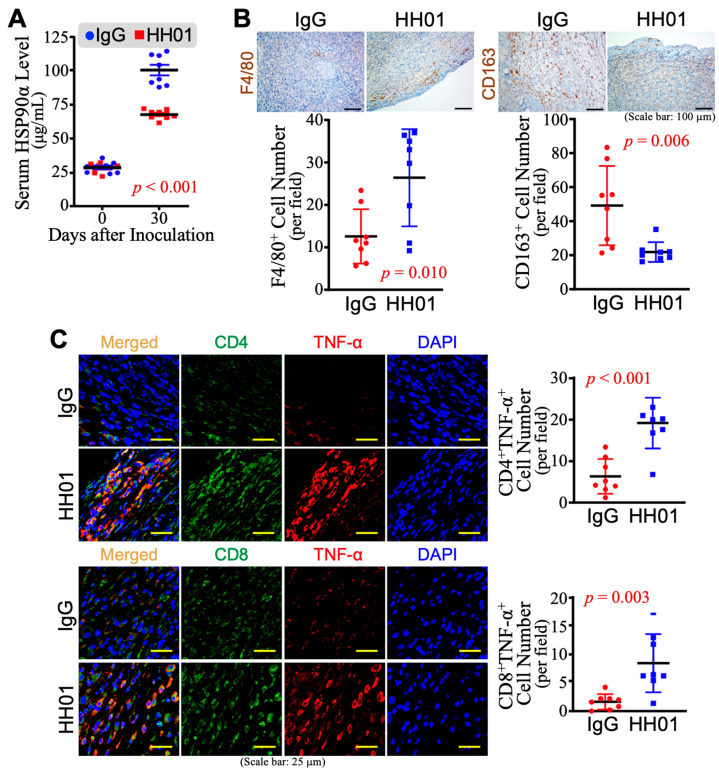
Immune reinvigoration of tumor microenvironment after HH01 treatment. (**A**) Serum HSP90α levels of experimental mice. HH01 treatments significantly suppressed the elevation of serum HSP90α levels caused by the tumor growth of Panc 02 plus EndoMT cell grafts. (**B**) Anti-F4/80 and anti-CD163 immunohistochemical staining analyses of the described tumors. HH01 treatments resulted in a significantly higher average level of F4/80^+^ cells (pan-macrophages) but a significantly decreased average level of CD163^+^ cells (M2-macrophages) in the tumors. (**C**) Anti-CD4, anti-CD8, and anti-TNF-α immunohistofluorescent staining analyses of the described tumors. HH01 treatments resulted in significantly higher average levels of CD4^+^TNF-α^+^ cells (effective T-cells) and CD8^+^TNF-α^+^ cells (cytotoxic T-cells) in the tumors.

To investigate if the suppression of tumor growth by HH01 was correlated with the reduction of serum HSP90α levels, mouse serum samples were collected from mice treated with IgG or HH01 for further eHSP90α measurement. As shown in Figure 6A, HH01 treatment resulted in a significant decrease in average eHSP90α level in the mouse sera. Considering eHSP90α is a potent inducer of macrophage M2-polarization, we also noticed that HH01 treatment led to an obvious increase in tumor F4/80^+^ pan-macrophage levels but a reduction in CD163^+^ M2-macrophages, resulting in a significant decrease of the ratio of M2-macrophage to pan-macrophage (Figure 6B). Accordingly, both immune CD4^+^TNF-α^+^ effective T-cells and CD8^+^TNF-α^+^ cytotoxic T-cells were significantly increased upon HH01 treatment (Figure 6C). These data collectively suggest that the HH01 antibody is a potent agent for improving the immunity of the tumor microenvironment and suppressing EndoMT-assisted and M2-macrophage-involved tumor growth.

## 4. Discussion

**Figure 7:**
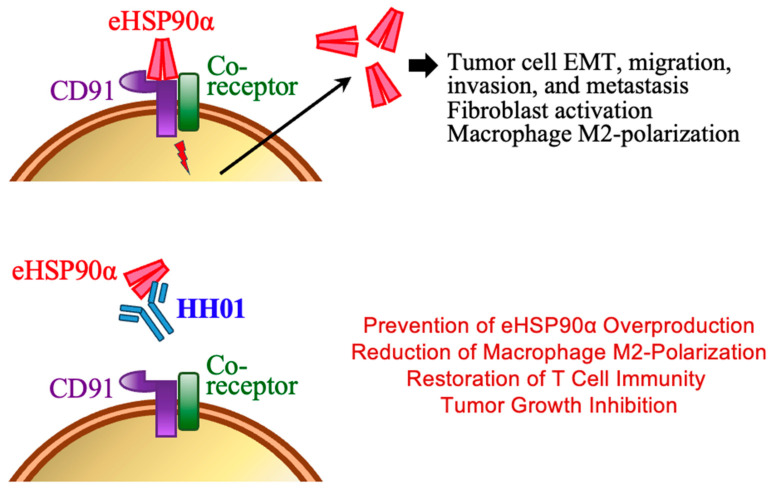
A schematic illustration summarizing our studies on the anti-PDAC mechanisms of HH01 antibody. eHSP90α, known as a tumor promoter, binds to the cell-surface receptor CD91 and associated co-receptor, facilitating tumor-promoting events such as neoplastic cell spreading, tumorigenic cell stemness, and macrophage M2-polarization. Moreover, eHSP90α sustains a feedforward loop of its production from macrophages and PDAC cells, among others, maintaining an immunosuppressive and proangiogenic eHSP90α-rich microenvironment that enhances tumor growth and malignant progression. HH01, a recombinant antibody, binds to the _235_AEEKEDKEEE_244_ and _251_ESEDKPEIED_260_ sites of eHSP90α, therefore blocking its ligation with CD91 and preventing downstream tumor-promoting functions. In mouse models, HH01 treatment suppresses EndoMT-derived CAFs-promoted tumor growth, eHSP90α overproduction, macrophage M2-polarization, and reinvigorates T-cell immunity in the tumor.

We have prepared a humanized anti-HSP90α antibody HH01 which can be potentially developed as a novel agent for suppressing PDAC, a cancer known for high mortality and extreme patient suffering. HH01 antibody has novel a.a. sequences in its CDRs and exhibits a high binding affinity towards HSP90α. It is modified from the mouse monoclonal antibody Clone-2 that arises by human full-length rHSP90α and can block eHSP90α binding to the cell-surface receptor CD91, therefore functionally inhibiting the downstream events including the excessive eHSP90α production induced by itself (Figure 7). eHSP90α is originally known as a tumor promoter by stimulating neoplastic cell EMT, migration, invasion, metastasis, and tumorigenic stemness [7,8,9,16]. In our in vitro anticancer assays, HH01 has shown the potency to suppress rHSP90α-induced cancer cell invasion and spheroid formation activities of PDAC and CRC cell lines. Furthermore, HH01 exhibits anticancer efficacies in mouse PDAC transplant models. It abolishes the tumor growth of PDAC cell grafts promoted by EndoMT-derived CAFs. In PDAC, 30~40% of CAFs can arise from the EndoMT of endothelial cells and contribute to PDAC desmoplasia [12]. Desmoplasia commonly occurs in malignancies, including PDAC and CRC, and is associated with tumor growth, immunosuppression, metastasis, and therapeutic resistance [22,23,24,25]. Our data suggest that targeting eHSP90α can be a novel strategy to suppress EndoMT-involved desmoplastic PDAC. It remains to be investigated whether HH01 also exhibits efficacies in mice with other desmoplastic cancers. The discovery of agents or strategies targeting desmoplasia may potentially alleviate the malignant progression and improve the therapeutic outcomes for desmoplastic cancer patients.

Clinically, a 5~10-fold increase in eHSP90α level has been detected in the serum specimens of PDAC patients [10]. In a normal physiological condition, HSP90α is primarily expressed and exerts its functions intracellularly. However, HSP90α can be secreted out of cells in an inflamed tissue microenvironment to facilitate tumor development and malignant progression. It has been observed that pancreas-infiltrating macrophages participate in the inflammatory reactions for PDAC formation and progression [10]. Macrophages can secrete OPN to stimulate endothelial cells to undergo EndoMT [16]. The resulting EndoMT-derived cells not only function as activated fibroblasts for extracellular matrix (ECM) production but also secrete some factors, especially eHSP90α, to induce the polarization of macrophages towards the M2 type [13]. These M2-type macrophages can secrete IL-10 and TGF-β to suppress immune T-cells, produce VEGF and bFGF to stimulate tumor angiogenesis, as well as express and secrete a great amount of eHSP90α to create an eHSP90α-rich tumor microenvironment. Therefore, this tumor microenvironment not only provides advantages for tumor growth and spreading but also sustains an immunosuppressive characteristic for the growing tumor. However, HH01 treatment can efficiently repress EndoMT-promoted tumor growth and serum eHSP90α levels and simultaneously alleviates macrophage M2-polarization and reinvigorates immune T-cells in the tumors. Unlike the agents that kill CAFs or deplete ECM components, the critical mode of action of HH01 is to interfere with the eHSP90α-CD91 ligation and thus inhibit the feedforward loop and excessive production of eHSP90α. Our data suggest that the HH01 antibody can be exploited to modulate the eHSP90α-rich tumor microenvironment and improve the immuno/chemotherapeutic outcomes for PDAC patients. We will evaluate the efficacies of combination therapies using HH01 antibody plus immuno/chemotherapeutic agents to suggest some novel therapeutic strategies for refractory desmoplastic cancers.

In this study, we have also determined that two HSP90α epitope regions, _235_AEEKEDKEEE_244_ and _251_ESEDKPEIED_260_, responsible for HH01 recognition, especially E237, E239, D240, K241, E253, and K255 were critical for binding to HH01. Furthermore, we have synthesized a peptide called P-46 with a sequence of the a.a. 227~272 region of HSP90α. P-46 could competitively suppress full-length eHSP90α-induced PDAC cell invasion and spheroid formation. The data are consistent with the previous literature result that the region a.a. 236~262 of HSP90α has an inhibitory effect on cell migration [26]. We therefore suggest that P-46 stands for an eHSP90α region, which is at least partly involved in the binding to CD91. As for eHSP90, the initial finding indicated that only HSP90α but not HSP90β was secreted into the culture medium of HT-1080 fibrosarcoma cells [27]. Furthermore, non-cancer functional studies indicated that human rHSP90α but not rHSP90β exhibited activities to stimulate human skin cell migration [28,29] and wound healing in pig skin models [29]. It is known that HSP90α performs a chaperone function, depending on the ATPase activity of the N-terminal domain. However, point mutations at the ATPase domain, HSP90α-E47A and HSP90α-D93N, conferred a loss of ATPase activity but did not alter the skin cell migration-promoting function [28]. A series of deletion mutations had further narrowed down that the 115-a.a. region (a.a. 236~350, called F-5 fragment) was responsible for the cell migration-promoting activity of eHSP90α [30]. In cancer cell studies, full-length rHSP90α acted as a tumor promoter by inducing cancer cell EMT, migration, invasion, and metastasis [7,8,9]. When MDA-MB-231 breast cancer cells were subjected to HIF-1α knockdown and thus lacked HSP90α secretion, the F-5 fragment could, as expected, restore the cell migration and invasion activities as effectively as full-length rHSP90α [5]. Specifically, rHSP90α but not rHSP90β treatment acted through an ATPase-independent manner to restore the defective tumorigenicity and metastasis of HSP90α-knockout MDA-MB-231 breast cancer cells [26]. A series of point mutations had suggested that K270 and K277 in the F-5 region were two critical a.a. for eHSP90α to exert the protumor functions. In addition, an anti-HSP90α monoclonal antibody 1G6-D7 had been developed to exhibit potent preventive efficacy against the tumor growth of native MDA-MB-231 cell grafts [26]. The epitope of the 1G6-D7 antibody had further been mapped to the K270 and K277-adjacent _293_TKPIWTRNP_301_ in the F-5 region. However, binding to the F-5 region is not a necessary condition for other agents that can suppress the protumor functions of eHSP90α. For example, the cell-impermeable small-molecule inhibitor DMAG-N-oxide inhibited the cell migration stimulated by full-length eHSP90α but not by the F-5 fragment. It is thought that the binding of DMAG-N-oxide to the N-terminal ATPase domain of full-length eHSP90α would cause a protein conformational change and thus disadvantage the eHSP90α binding to the cell-surface receptor CD91 [31].

HH01 antibody also has other superior characteristics that make it a novel anticancer agent. Unlike small-molecule HSP90α inhibitors, it does not exhibit toxicity to non-tumorous retinal pigment epithelial cells, and its multiple intravenous injections do not result in splenic enlargement in experimental mice. HH01 is quite water-soluble but not easily excreted out of mouse bodies, with a half-life of >18 days in the circulatory system. In conclusion, HH01 is a humanized anti-HSP90α antibody with novel CDRs and exhibits a high binding affinity towards HSP90α. By blocking the ligation of eHSP90α with the cell-surface receptor CD91, HH01 can further stop the de novo production of excess eHSP90α and thus achieve its pronounced anti-eHSP90α effect. HH01 also exhibits its superiority in anticancer functions. It effectively suppresses eHSP90-induced invasive and spheroid-forming activities in PDAC and CRC cell lines. It also potently restricts EndoMT-promoted tumor growth and restores tumor immunity. Mouse serum eHSP90α levels are also suppressed as well to reflect the HH01 anticancer efficacy. HH01 antibody is not easy to form aggregate. It is quite water-soluble but not easily excreted out of mouse bodies with a T_1/2_ of >18 days in blood. It is not cytotoxic to retinal pigmented epithelial cells and has no obvious toxic effect on mouse organs. Our studies suggest that HH01 can be potentially developed as an effective agent against desmoplastic PDAC.

## Data Availability

All data generated or analyzed during this study are included in this published article.
